# Assessing Orthorexia Nervosa Among University Students: An Observational Study Analyzing Prevalence and Psychological Characteristics

**DOI:** 10.3390/nu17132078

**Published:** 2025-06-23

**Authors:** Rosanna Sanseverino, Sara Guidotti, Carlo Pruneti

**Affiliations:** Clinical Psychology, Clinical Psychophysiology, and Clinical Neuropsychology Laboratories, Department of Medicine and Surgery, University of Parma, 43126 Parma, Italy; sara.guidotti@unipr.it (S.G.); carlo.pruneti@unipr.it (C.P.)

**Keywords:** orthorexia nervosa, prevalence, university students, feeding and eating disorders, eating disorders, healthy eating

## Abstract

Background/Objectives: The prevalence of orthorexia nervosa (ON) is increasing over time. Additionally, specific social categories seem to be more affected. In the literature, the prevalence of university students suffering from ON is unclear, ranging from 7% to 83%. Nonetheless, ON shares pathological traits with both eating and obsessive–compulsive disorders, making its etiology and therapeutic perspectives complex. This study aimed to investigate the prevalence of ON and explore its psychological characteristics in a sample of university students. Methods: A total of 205 students from the University of Parma were consecutively recruited using a convenience sampling procedure. Participants completed the Orthorexia Nervosa Questionnaire-15 (ORTO-15) to assess ON, the Eating Disorder Inventory-3 (EDI-3) to investigate eating behavior, the Symptom Checklist-90-Revised (SCL-90-R) to detect psychological symptoms, and the P Stress Questionnaire (PSQ) to describe stress-related lifestyle. Based on the scores obtained on the ORTO-15, a group of orthorexic students (ORTO-15 score ≤ 35) was compared with a group of non-orthorexic students (ORTO-15 score > 35). Results: The prevalence of university students with ON was nearly 42% (specifically, 41.95%). Furthermore, orthorexic students reported significantly higher levels of emotional dysregulation, perfectionism, and asceticism on the EDI-3 as well as affective problems and overcontrol in general. Furthermore, although there were no differences between the groups regarding psychological symptoms, an increase in sense of responsibility, vigor, and hyperactivity, as well as decreased free time on the PSQ, characterized the orthorexic student group. Conclusions: The results support that orthorexia nervosa emerged as a concerning phenomenon among university students, with increasing evidence pointing to its psychological correlates. Nonetheless, the fact that ON shares psychological characteristics with eating disorders highlights the clinical importance of implementing multidimensional assessments and multidisciplinary therapeutic approaches for individuals presenting with orthorexic-type eating behavior disorders.

## 1. Introduction

According to the World Health Organization (WHO), health is “a state of complete physical, mental, and social well-being and not merely the absence of disease or infirmity” [1]. In this framework, nutrition emerges as a key determinant of both physical and mental health. A balanced diet represents an effective tool not only for the prevention of numerous diseases, but also for the management and treatment of many health conditions. To illustrate, healthy eating habits are crucial in preventing overweight and obesity, which remains a major public health concern worldwide [2].

Although the pursuit of healthy eating is crucial, it may lead to an emerging condition known as orthorexia nervosa (ON) when obsessive and rigid [3]. ON shares traits of rigidity and perfectionism with obsessive–compulsive disorder (OCD), as comorbidity between the two conditions is frequently reported [4]. However, although there was an overlap between ON and OCD-related symptoms, Donini and colleagues classified ON as an eating disorder (ED) [5]. The reason lies in the fact that strict adherence to self-imposed dietary rules is observed in individuals with anorexia nervosa (AN). More specifically, while such rules in AN are aimed at weight loss and the pursuit of thinness, the declared goal in ON is the maintenance or improvement of health, whether real or perceived. Nevertheless, the only rigid adherence to dietary rules is not a sufficient condition to diagnose ON, as clinical impairment—either physical or psychosocial—is also required [5]. By way of illustration, individuals with ON may avoid social occasions involving food to remain faithful to their dietary regime [6]. This kind of behavior can negatively impact both physical and mental health, similarly to what is observed in AN as well as bulimia nervosa (BN) [7].

The avoidance of specific food groups or cooking methods due to fears about their impact on health is typical of ON, which may also resemble the diagnostic features of avoidant–restrictive food intake disorder (ARFID), as described in the DSM-5-TR [8]. Both in ON and ARFID, restrictive eating patterns can lead to weight loss and nutritional deficiencies as well as social impairment. However, the nature of the underlying fears differs because individuals with ARFID tend to fear immediate consequences of food intake (e.g., choking), whereas those with ON are more concerned about long-term health risks (e.g., cardiovascular disease) [5].

Among younger populations, attention to food choices is becoming increasingly central. Many young people engage in restrictive diets at an early age, which is considered a risk factor for the development of EDs [7].

Previous studies on ON reported considerably variable prevalence rates, ranging from 6.9% (in the general population) to over 80% (in nutrition students), depending on the diagnostic criteria and assessment tools used [6]. Specifically taking into account the Italian population, the calculated range varies from 6.9% to over 60%, with differences attributable to the instruments and cut-offs used [9,10]. A review by Costa et al. [11] emphasized the lack of validated diagnostic criteria and the variability in assessment tools —especially concerning the ORTO-15. These inconsistencies hindered the determination of ON’s true prevalence and its recognition as a distinct ED, until now.

An interesting study was conducted by Dell’Osso et al. at the University of Pisa in 2016 [12]. The authors calculated the prevalence and specific characteristics of orthorexic behaviors in samples of young Italian adults (2130 students and 696 university employees), detecting a frequency of 32.7%, using the 35-score threshold of the ORTO-15 questionnaire. Involving only a sample of university students in a subsequent 2018 study, the prevalence of ON further increased to 34.9% [13].

From our previous data published in 2024 [14], the prevalence of ON in a sample of 165 students from the University of Parma was equal to 83%, using the 40-score threshold of the ORTO-15. Using the 35-score threshold in a smaller sample size of a pilot study, the percentage dropped to 38.10% [15], which is coherent with the findings described by the researchers of Pisa, still highlighting an increase in the last 7 years.

Another Italian study reported a prevalence of approximately 25% in a university sample primarily composed of students from dietetics and exercise and sport sciences, with additional representation from biology courses [16]. The authors attributed the high prevalence of orthorexic tendencies to the increasing emphasis on nutritional education among young adults in recent years.

Similar research on university populations was conducted in Spain, detecting a prevalence of 1,5%. These data can highlight how prevalence varies greatly depending on the instrument used and the geographic area [17].

In light of the evidence described, we aimed to enrich the existing literature on the prevalence of ON, calculating it on a larger sample of students from the University of Parma. Nevertheless, we aimed to contribute to the ongoing debate on the classification of ON as an ED or as a condition related to OCD, exploring the clinical characteristics concerning eating behavior, psychological symptoms, and lifestyle of a group of orthorexic students compared to controls.

## 2. Materials and Methods

### 2.1. Participants

This cross-sectional study involved 205 students from the University of Parma, recruited through convenience sampling. The inclusion criteria were as follows: (1) age ≥ 18 years; (2) provision of informed consent; and (3) absence of psychiatric and/or neurological conditions (e.g., history of head trauma, epilepsy) or physical impairments (e.g., sensory deficits, such as visual or hearing disturbances) that could interfere with the administration of the psychological questionnaires.

### 2.2. Procedure

Participants were recruited through an invitation disseminated via the university’s official mailing list. Those who expressed interest received a brief overview of the study’s aims and were invited to the Clinical Psychology, Clinical Psychophysiology, and Clinical Neuropsychology Laboratories at the Department of Medicine and Surgery of the University of Parma.

The experimental procedures were under the 2024 Declaration of Helsinki of the World Medical Association and the 2005 Universal Declaration on Bioethics and Human Rights of UNESCO.

### 2.3. Measures

The standardized self-report questionnaire Orthorexia Nervosa Questionnaire-15 (ORTO-15) was used to assess ON [9]. This instrument consists of 15 items, to which participants respond using a 4-point Likert scale (“always”, “often”, “sometimes”, and “never”). The items are grouped into cognitive–rational, emotional, and clinical sub-scales. A total score is calculated by summing the values of the three sub-scales. The literature presents a higher (40-score threshold) and a lower cut-off (35-score threshold) [11], with a higher sensitivity for the first reference and a higher specificity for the second one. In the present study, the more restrictive cut-off of 35 was used, since it increases the diagnostic accuracy of the questionnaire, improving its ability to distinguish between individuals with pathological tendencies and individuals without them, and reducing false positives [10].

The Eating Disorder Inventory-3 (EDI-3) [18], Italian adaptation [19], was used to investigate the eating behavior. This is a self-report questionnaire composed of 91 items rated on a 6-point Likert scale, from “never” to “always”. It evaluates psychological traits and symptoms related to eating disorders across 12 primary scales: three eating disorder-specific scales—Drive for Thinness (DT), Bulimia (B), and Body Dissatisfaction (BD)—and nine general psychological scales—Low Self-Esteem (LSE), Personal Alienation (PA), Interpersonal Insecurity (II), Interpersonal Alienation (IA), Interoceptive Deficits (IDs), Emotional Dysregulation (ED), Perfectionism (P), Asceticism (A), and Maturity Fears (MFs). Composite scores are derived by combining specific scales: the Eating Disorder Risk Composite (EDRC; DT + B + BD) reflects the overall risk of eating disorders; the remaining composites assess broader psychological constructs: Inadequacy Composite (IC; LSE + PA), Interpersonal Problems Composite (IPC; II + IA), Affective Problems Composite (APC; ID + ED), Overcontrol Composite (OC; P + A), and the General Psychological Maladjustment Composite (GPMC; sum of the nine psychological scales). Raw scores are converted into percentile ranks. A score above the 70th percentile is considered indicative of a clinically relevant profile. The internal consistency of the EDI-3 scales, as assessed by Cronbach’s alpha, ranged from 0.70 to 0.94.

The Symptom Checklist 90-Revised (SCL-90-R) [20] is a 90-item self-report questionnaire designed to assess a broad range of psychological symptoms experienced in the past week. Items are rated on a 5-point Likert scale ranging from 0 (“not at all”) to 4 (“extremely”). The instrument includes nine clinical dimensions: Somatization (SOM), Obsessive–Compulsive (O–C), Interpersonal Sensitivity (I-S), Depression (DEP), Anxiety (ANX), Hostility (HOS), Phobic Anxiety (PHOB), Paranoid Ideation (PAR), and Psychoticism (PSY). For each dimension, a mean score is calculated by dividing the total raw score by the number of scale items. A global index, the Global Severity Index (GSI; α = 0.87), represents the respondent’s overall level of psychological distress and is computed by averaging all 90 items. The authors assert that T-scores ≥ 63 in two or more clinical scales or the GSI scale suggest the presence of a clinically significant state of distress, satisfying the so-called caseness criterion [21]. Internal consistency coefficients (Cronbach’s α) for the symptom dimensions range from 0.67 (PHOB) to 0.87 (DEP), proving acceptable to excellent reliability.

The P Stress Questionnaire (PSQ) [22] is a self-administered questionnaire consisting of 32 forced-choice items: 16 require a dichotomous yes/no response, 15 offer three options (often/sometimes/never), and 1 compares personal behavior to that of the general population (definitely/not more than others). The questionnaire assesses six main dimensions: Sense of Responsibility (SR), assessing the tendency to take life too seriously; Vigor (V), reflecting perceived vitality and energy in coping with stress; Stress Disorders (SDs), identifying symptoms related to stress responses; Precision and Punctuality (PP), evaluating tendencies toward meticulousness and persistence; Leisure Time (LT), indicating difficulties in relaxation; and Hyperactivity (I), linked to beliefs about endurance to fatigue. For each factor, raw scores are standardized into nine-point stanine scales, where scores above 7 are considered high, scores below 4 low, and scores between 4 and 7 average. A total score (TOT) is also available, which summarizes the general propensity to adopt a lifestyle that predisposes to stress-related physical disorders.

### 2.4. Statistical Analysis

SPSS software was used to perform statistical analyses (version 28.0.1.0; IBM Corp, Armonk, NY, USA). The percentage of people exceeding the cut-off of 35 on the ORTO-15 scale was calculated.

After checking that the assumptions of normality for performing parametric statistics were respected, the mean (M) and standard deviation (SD) of each score of the questionnaires’ scales were calculated.

Differences between groups based on sociodemographic characteristics (i.e., age, sex, marital status, and education level) and clinical characteristics (i.e., SCL-90-R and PSQ scores) were calculated using the Chi-square test and the T-test for independent samples.

## 3. Results

The sample had a mean age of 25 ± 5 years and consisted of 55.66% females and 44.4% males. Participants were enrolled in a wide range of degree programs offered by the University of Parma, with a higher prevalence of students from health sciences, nutrition, and exercise science courses. Most of the participants were single (192 out of 205; 93.7%), and only a minority were married or cohabiting (13 out of 205; 6.3%). The majority were full-time students (186 out of 205; 90.7%), while a smaller proportion were also employed (19 out of 205; 9.3%). Regarding educational level, 41.0% (84 out of 205) held a high school diploma, while 59.0% (121 out of 205) had already obtained a university degree.

First, the ORTO-15 cut-off (set at ≤ 35) was used to identify a group of university students with ON. The group of participants with a score ≤ 35 comprised 86 people, representing 41.95% of the total sample.

Concerning sociodemographic variables, the group of students with ON differed from the control group only for gender (Table 1).

No significant differences between groups were observed looking at the risk scales of the EDI-3. Regarding the composite scales, significantly higher scores were observed for orthorexic students in the APC and OC, but significantly lower in the IPC. Looking at the clinical scales, students in the orthorexic group obtained significantly higher scores in the ED, P, and A scales. On the contrary, the II scores were significantly higher in the control group (Table 2).

Looking at psychological symptoms, no significant differences emerged between the groups (Table 3). However, both orthorexic and non-orthorexic students manifested elevated levels of psychological symptoms, with mean T-scores exceeding the clinical cut-off for Somatization, Depression, Paranoid Ideation, and Psychoticism. Furthermore, this trend was observed for the GSI score, suggesting a generally high level of mental distress in the overall sample.

Lastly, the comparison between the groups regarding the PSQ sub-scale scores revealed higher scores of Sense of Responsibility, Vigor, and Hyperactivity in orthorexic students. Nevertheless, the total PSQ score was also significantly different between the groups (Table 4).

## 4. Discussion

A preliminary study conducted in 2004 assessed the prevalence of ON in the general Italian population. Among the 404 individuals examined, 6.9% were positive for ON [23]. However, subsequent research focusing on young adults or university student populations reported significantly higher prevalence rates [13]. The present study found a prevalence of 42% using the 35-score threshold, which is in line with previous data. Although there are two cut-off values (i.e., 40-score and 35-score), the cut-off ≤ 35 proved a higher specificity (94.2 vs. 75.8%) as well as the highest negative predictive value (91.1%) [13]. In any case, the 42% of ON is an alarming datum, which exceeds the 37% calculated in a group of students at the University of Pisa in 2018 [13] and documented a constant increase over time.

This high prevalence may be partially attributable to the academic background of the participants. Approximately half of the students involved in the study were enrolled in health-related degree programs, which emphasize topics including nutrition, physical activity, and overall health. [16] By way of illustration, the findings of Korinth et al. [24] evidenced that nutrition students tend to reduce their caloric intake more significantly than control group students, progressively improving their dietary habits throughout the academic training. Donini et al. [5] suggested that university programs, such as dietetics and nutrition, may be associated with an increased risk of developing ON.

First, ON was related to the sociodemographic data of the participants. Although the literature reports a significant association between ON and lower educational level [23,25,26], this aspect did not emerge from our data. On the other hand, a higher prevalence of males in the sample of orthorexic students was found. These data, although at the threshold of statistical significance, are in line with what was found by other research groups but in contrast with other studies. An interesting 2023 review by Atzeni et al. [27] noted that eight out of 14 studies investigating gender differences excluded its influence on ON. Other studies reported a gender-related association, sometimes female (three out of the six remaining studies) but sometimes male (three out of the six remaining studies). The gender distribution in our sample highlighted a prevalence of males, in any case with a significantly lower ratio than that of AN and BN (2:1 vs. 10:1, respectively) [10].

Therefore, the orthorexic group was compared with the control one to find differences in the EDI questionnaire scores. Consistent with previous research on EDs, which identifies emotional dysregulation as a transdiagnostic factor across all EDs [28], our findings confirmed that individuals with ON exhibit significantly higher levels of emotional dysregulation as well. This result may further support the hypothesis that ON shares core psychological traits with traditional EDs. Similarly to individuals with AN, orthorexic individuals might use rigid dietary control as a maladaptive strategy to manage negative affect [29]. The elevated scores observed on the Overcontrol Composite (OC) scale in the orthorexic group seem to support the psychological interpretation that links ON to maladaptive coping strategies [30]. Perfectionism emerged as a salient psychological factor in the literature as well [31]. In EDs, particularly in AN, individuals often impose excessively high standards related to physical appearance and performance, with self-esteem and identity strongly tied to body image [32]. Similarly, elevated levels of perfectionism in the orthorexic group may suggest that thoughts and behaviors might be driven by a desire to adhere to an ideal diet, pursued with rigid and inflexible rules [33]. It has been documented that ON individuals often believe that healthy eating enhances their physical appearance, and strict compliance with their dietary rules may boost their self-esteem [5].

Another relevant aspect related to the EDI-3 clinical scale regards asceticism, which refers to the tendency to control physical needs and impulses through self-denial and suffering [18]. For instance, asceticism typically manifests in AN, suppressing hunger and fasting behaviors [34]. Among individuals at risk for ON, the avoidance of foods perceived as unhealthy might occur, as well as the perception of dietary “transgressions” that lead to negative emotional consequences, such as guilt [35]. As a result, individuals with ON might engage in compensatory behaviors (i.e., fasting, excessive exercise, or detox routines) to alleviate feelings of anxiety, guilt, or impurity, as verified by Meyer and colleagues [36], who underlined that compulsive exercise might serve as a dysfunctional emotional regulation strategy among ED populations.

To deeply explore obsessive and compulsive tendencies, the SCL-90-R was administered as well. No significant differences in all scales were found, unlike what was expected based on pre-existing scientific literature, as obsessive–compulsive symptoms are a frequent comorbidity of ON [4]. However, it should be noted that several clinical scales were above the 63 T-score cut-off, confirming a generally high level of mental distress in the selected population [37].

Still, concerning the EDI-3 questionnaire, the control group scored higher on the Interpersonal Insecurity (II) scale. Although this finding is difficult to interpret, it has been hypothesized that a response bias related to self-perception may exist among orthorexic students. Self-report questionnaires reflect subjective experience, and it is plausible that perfectionistic self-demands and a heightened sense of control may contribute to a perceived sense of efficacy and security in interpersonal contexts. Prior research goes in this direction, proving that perfectionism in students, when coupled with adequate emotional regulation, enhances academic control and self-management [38]. However, in the presence of emotional dysregulation, as already observed in ON and EDs, perfectionism might lead to distress [32].

Looking at the PSQ scores, orthorexic students had higher levels of sense of responsibility, perceived vigor, and hyperactivity, and a lower ability to benefit from spare time. These data confirm the results of our previous work [14] and deserve a reflection based on what has been found in the literature. Although ON has been associated with an increase in time dedicated to aerobic physical activity and a greater dependence and compulsivity to physical exercise [39], the absence of the ability to enjoy free time could mean that these individuals dedicate themselves to leisure activities, but applying the same mental rigidity that does not allow psychophysical recovery.

Another recent Italian study [40] reported associations between orthorexic tendencies and BMI, adherence to dietary plans, and physical activity. Taken together, the findings from this and our previous studies underscore the importance of a multidimensional assessment, that includes psychological, psychophysiological, and behavioral characteristics. Moreover, from a clinical standpoint, the recovery process should be supported by a multidisciplinary team.

Although this research provides new insights into the characteristics and prevalence of ON, it is not without limitations. First, the sample size was relatively small and consisted primarily of university students, which limits the generalizability of the findings. Future studies could benefit from larger and more diverse samples, potentially recruited through online platforms, to include a broader demographic. It is plausible that in a more heterogeneous population, significant differences in obsessive–compulsive traits, reported in previous literature [5], might emerge. Moreover, the present sample was a convenience sample. Despite dissemination through the University of Parma’s official newsletter, not all degree programs were equally represented. Lastly, due to the cross-sectional and observational nature of the study, causal relationships between variables cannot be established. In addition, future studies should include other measures related to lifestyle (i.e., body mass index, physical activity, previous obesity, etc.).

We believe that future studies should be implemented to enrich the literature on the topic. The clinical implications of this type of study are numerous. In the broad spectrum of EDs, ON emerges as a growing public health concern. Eating behavior and weight management in different age groups and social categories (i.e., specific clinical populations, such as diabetes, chronic gastrointestinal diseases, etc.) represent apparently antithetical conditions, but they coexist in the same sociocultural landscape. Especially considering the group of young adults and, specifically, university students, the association between nutrition, body image, and health represents an urgent problem due to the lack of primary and secondary prevention programs. Prevention programs in public health should find a delicate balance between the promotion of healthy eating, especially starting from developmental age, and the prevention of EDs, whose age of onset is becoming increasingly early. Just to illustrate the issue, data from the latest national mapping of EDs conducted in public health centers in 2022 determined that 59% of patients are between 13 and 25 years old, and 6% are under 12 years old [41].

From a clinical perspective, these results urge institutions to create guidelines for prevention at various levels (i.e., primary, secondary, and tertiary), identifying the population groups most at risk and providing psychoeducation programs. The significant associations between ON and clinical characteristics of EDs, as well as with the lifestyle that predisposes to stress-related disorders, underline the involvement of different areas of functioning of the person. In other words, ON determines an alteration of eating behavior, which is supported by traits of mental suffering and followed by dysfunctional behaviors. A complete and multidimensional evaluation is recommended for individuals at risk of ON, who can benefit from the support provided by a multidisciplinary team. The synergic work between psychologists and nutritionists is indicated to promote good adherence to a healthy diet but also supported by a lifestyle appropriate to the psychophysical well-being of individuals.

## 5. Conclusions

The present study aimed to investigate the prevalence and psychological characteristics of ON in a sample of university students. The findings suggest that individuals with ON share several traits with those affected by EDs, including emotional dysregulation, perfectionism, asceticism, and overcontrol. A heightened sense of responsibility, vigor, and hyperactivity was also observed, along with difficulties in benefiting from leisure time.

These conclusions, supported by our results, highlight the need to support and encourage psychological interventions tailored to university students, especially those who tend to regulate their emotions through dysfunctional behaviors, such as those characterized by control over food. Some groups of students could benefit from learning enhanced emotional self-regulation strategies, and the repercussions could be evident both on the psychological and physical level.

Nevertheless, clinical psychology research should aim to intercept the often implicit “call for help”, especially in cases where individuals experience psychological distress but do not seek professional assistance, potentially due to persistent social stigma, engaging in dysfunctional coping styles.

In this regard, universities play a central role for the student population. Psychological services are limited in availability in educational institutions, and the limited resources do not allow for an adequate functional analysis of the behaviors used by students to manage stress.

## Figures and Tables

**Table 1:** Comparison of sociodemographic characteristics between the non-orthorexic and the orthorexic students.

Variable	ORTO-15 Score > 35 (*n* = 119)	ORTO-15 Score ≤ 35 (*n* = 86)	t or χ^2^	*p*
Age (years), *M* (*SD*)	25.05 (5.64)	25.22 (5.60)	t (204) = −0.21	0.42
Gender, *N* (%)			χ^2^ (1, *N* = 205) = 3.80	0.05
Male	46 (38.7%)	45 (52.3%)		
Female	73 (61.3%)	41 (47.7%)		
Occupation, *N* (%)			χ^2^ (1, *N* = 205) = 0.25	0.62
Student	109 (91.6%)	77 (89.5%)		
Student + worker	10 (8.4%)	9 (10.5%)		
Education level, *N* (%)			χ^2^ (1, *N* = 205) = 0.05	0.83
High school graduation	48 (40.3%)	36 (41.9%)		
University degree	71 (59.7%)	49 (58.1%)		
Marital status, *N* (%)			χ^2^ (1, *N* = 205) = 0.10	0.75
Unmarried	112 (94.1%)	80 (93.0%)		
Married/cohabitant	7 (5.9%)	6 (7.0%)		
Children, N (%)			χ^2^ (1, *N* = 205) = 0.72	0.40
No	115 (96.6%)	81 (94.2%)		
Yes	4 (3.4%)	5 (5.8%)		

**Table 2:** Comparison of percentile scores on the EDI-3 between the non-orthorexic and the orthorexic students.

	ORTO-15 Score > 35 (*n* = 119)		ORTO-15 Score ≤ 35 (*n* = 86)		t (204)	*p*	D
	M	SD	M	SD			
EDI-3 Risk Scales							
Drive for Thinness	32.40	28.14	39.13	29.92	−1.59	0.06	−0.23
Bulimia	34.93	31.31	38.21	30.80	−0.72	0.24	−0.11
Body Dissatisfaction	36.23	24.38	36.03	27.02	−0.06	0.48	−0.01
EDI-3 Clinical Scales							
Low Self-Esteem	43.75	28.01	41.14	26.86	0.65	0.26	0.10
Personal Alienation	45.01	29.18	43.87	27.45	0.27	0.39	−0.04
Interpersonal Insecurity	51.35	29.17	43.62	28.33	1.83	0.04	0.27
Interpersonal Alienation	47.13	27.38	42.94	27.94	1.04	0.15	0.15
Interoceptive Deficit	47.72	29.01	51.96	27.85	−1.07	0.16	−0.15
Emotional Dysregulation	39.35	28.70	47.03	30.71	−2.04	0.02	−0.30
Perfectionism	44.83	30.55	54.87	27.63	−2.33	0.01	−0.34
Ascetism	42.97	27.10	51.26	25.10	−2.12	0.02	−0.31
Fear of Maturity	48.86	31.50	49.85	31.11	−0.21	0.42	−0.03
EDI-3 Composite Scales							
Eating Disorder Risk Composite	35.09	24.98	38.85	27.24	−0.99	0.16	−0.15
Ineffectiveness Composite	45.53	27.67	43.72	25.93	0.46	0.32	−0.07
Interpersonal Problems Composite	51.09	27.89	44.29	27.45	1.67	0.05	0.25
Affective Problems Composite	45.28	26.69	52.88	26.09	−1.96	0.030	−0.29
Overcontrol Composite	44.53	27.40	55.45	26.00	−2.77	0.0030	−0.41
General Psychological Maladjustment Composite	48.31	26.18	51.33	25.24	−0.80	0.21	−0.12

**Table 3:** Comparison of the SCL-90-R T-scores between the non-orthorexic and the orthorexic students.

	ORTO-15 Score > 35 (*n* = 119)		ORTO-15 Score ≤ 35 (*n* = 86)		t (204)	*p*	D
	M	SD	M	SD			
Somatization	56.69	12.96	53.37	15.24	−1.33	0.09	−0.19
Obsessive–Compulsive	67.07	16.30	69.45	16.19	−1.01	0.16	−0.15
Interpersonal Sensitivity	62.87	19.50	63.81	17.65	−0.35	0.36	−0.05
Depression	63.24	17.29	64.94	16.90	−0.69	0.25	−0.10
Anxiety	60.83	17.70	63.77	19.11	−1.12	0.13	−0.16
Hostility	57.00	13.97	57.31	13.74	−0.15	0.44	−0.02
Phobic Anxiety	55.27	17.96	55.74	15.06	−0.19	0.42	−0.03
Paranoid Ideation	64.60	18.02	66.29	18.20	−0.65	0.26	−0.09
Psychoticism	65.72	21.10	70.09	25.08	−1.33	0.09	−0.19
General Severity Index	64.83	16.93	67.58	17.40	−1.19	0.13	−0.16

**Table 4:** Comparison of the PSQ raw scores between the non-orthorexic and the orthorexic students.

	ORTO-15 Score > 35 (*n* = 119)		ORTO-15 Score ≤ 35 (*n* = 86)		t (204)	*p*	D
	M	SD	M	SD			
Sense of Responsibility	5.78	3.38	7.23	2.93	−3.15	**<0.0010**	**−0.45**
Vigor	2.87	2.40	3.54	2.19	−2.01	**0.02**	**−0.21**
Stress Disorders	3.18	1.81	3.39	1.61	−0.85	0.20	−0.12
Precision and Punctuality	3.81	1.94	3.65	1.96	0.56	0.29	0.08
Spare Time	2.11	2.00	1.65	1.90	1.63	**0.05**	**0.23**
Hyperactivity	5.03	1.47	5.39	1.61	−1.63	**0.05**	**−0.23**
Total Score	25.41	8.15	28.04	6.49	−2.45	**0.008**	**−0.35**

## Data Availability

The dataset is available upon request from the authors. The raw data supporting the conclusions of this article will be made available by the authors on request.

## References

[1] World Health Organization (1948). Preamble to the Constitution of the World Health Organization as Adopted by the International Health Conference, New York, 19–22 June, 1946.

[2] Organizzazione Mondiale della Sanità Healthy Diet 29 April 2020. https://www.who.int/news-room/fact-sheets/detail/healthy-diet.

[3] Bratman S. (1997). Health Food Junkie: Obsession with Dietary Perfection Can Sometimes Do More Harm than Good, Says One Who Has Been There. Yoga J..

[4] Duradoni M., Gursesli M.C., Fiorenza M., Guazzini A. (2023). The Relationship between Orthorexia Nervosa and Obsessive Compulsive Disorder. Eur. J. Investig. Health Psychol. Educ..

[5] Donini L.M., Barrada J.R., Barthels F., Dunn T.M., Babeau C., Brytek-Matera A., Cena H., Cerolini S., Cho H.H., Coimbra M. (2022). A consensus document on definition and diagnostic criteria for orthorexia nervosa. Eat. Weight Disord. EWD.

[6] Dunn T.M., Bratman S. (2016). On orthorexia nervosa: A review of the literature and proposed diagnostic criteria. Eat. Behav..

[7] Berner L.A., Crosby R.D., Cao L., Engel S.G., Lavender J.M., Mitchell J.E., Wonderlich S.A. (2017). Temporal associations between affective instability and dysregulated eating behavior in bulimia nervosa. J. Psychiatr. Res..

[8] American Psychiatric Association (2022). Diagnostic and Statistical Manual of Mental Disorders.

[9] Donini L.M., Marsili D., Graziani M.P., Imbriale M., Cannella C. (2005). Orthorexia nervosa: Validation of a diagnosis questionnaire. Eat. Weight Disord.

[10] Ramacciotti C.E., Perrone P., Coli E., Burgalassi A., Conversano C., Massimetti G. (2011). Orthorexia nervosa in the general population: A preliminary screening using a self-administered questionnaire (ORTO-15). Eat. Weight Disord..

[11] Costa C.B., Hardan-Khalil K., Gibbs K. (2017). Orthorexia Nervosa: A Review of the Literature. Issues Ment. Health Nurs..

[12] Dell’Osso L., Abelli M., Carpita B., Massimetti G., Pini S., Rivetti L., Gorrasi F., Tognetti R., Ricca V., Carmassi C. (2016). Orthorexia nervosa in a sample of Italian university population. Riv. Psichiatr..

[13] Dell’oSso L., Carpita B., Muti D., Cremone I.M., Massimetti G., Diadema E., Gesi C., Carmassi C. (2018). Prevalence and characteristics of orthorexia nervosa in a sample of university students in Italy. Eat. Weight Disord..

[14] Guidotti S., Fiduccia A., Murgolo M., Pruneti C. (2024). Comparison between physical activity and stress-related lifestyle between orthorexic and non-orthorexic university students: A case–control study. Nutrients.

[15] Guidotti S., Fiduccia A., Sanseverino R., Pruneti C. (2025). Multidimensional assessment of orthorexia nervosa: A case-control study comparing eating behavior, adherence to the Mediterranean diet, body mass index, psychological symptoms, and autonomic arousal. Nutrients.

[16] Bo S., Zoccali R., Ponzo V., Soldati L., De Carli L., Benso A., Fea E., Rainoldi A., Durazzo M., Fassino S. (2014). University courses, eating problems and muscle dysmorphia: Are there any associations?. J. Transl. Med..

[17] Manero-Higuera S., Garcés-Rimón M., Iglesias-López M.T., López-Moreno M. (2025). Orthorexia Nervosa: Prevalence Among Spanish University Students and Its Effects on Cardiometabolic Health. Nutrients.

[18] Garner D.M. (2004). Eating Disorder Inventory-3. Professional Manual. Lutz, FL: Psychological Assessment Resources. Int. J. Eat. Disord..

[19] Giannini M., Pannocchia L., Dalle Grave R., Muratori F., Viglione V. (2008). Adattamento italiano dell’EDI-3. Eating Disorder Inventory-3.

[20] Prunas A., Sarno I., Preti E., Madeddu F., Perugini M. (2012). Psychometric properties of the Italian version of the SCL-90-R: A study on a large community sample. Eur. Psychiatry.

[21] Derogatis L.R., Svitz K.L., Maruish M.E. (1999). The SCL-90-R, Brief Symptom Inventory, and Matching Clinical Rating Scales. The Use of Psychological Testing for Treatment Planning and Outcomes Assessment.

[22] Pruneti C. (2011). The P Stress Questionnaire: A new tool for the evaluation of stress-related behaviors. Eur. J. Clin. Psychol. Psychiatry.

[23] Donini L.M., Marsili D., Graziani M.P., Imbriale M., Cannella C. (2004). Orthorexia nervosa: A preliminary study with a proposal for diagnosis and an attempt to measure the dimension of the phenomenon. Eat. Weight. Disord. Stud. Anorex. Bulim. Obes..

[24] Korinth A., Schiess S., Westenhoefer J. (2010). Eating behaviour and eating disorders in students of nutrition sciences. Public Health Nutr..

[25] Depa J., Schweizer J., Bekers S.K., Hilzendegen C., Stroebele-Benschop N. (2017). Prevalence and predictors of Orthorexia Nervosa among germane students using the 21-item-DOS. Eat. Weight Disord..

[26] Malmborg J., Bremander A., Olsson M.C., Bergman S. (2017). Health status, physical activity, and orthorexia nervosa: A comparison between exercise science students and business students. Appetite.

[27] Atzeni E., Converso D., Loera B. (2020). L’ortoressia nervosa tra attenzione per la qualità dell’alimentazione e disturbi alimentari: Criteri diagnostici e strumenti di valutazione. Riv. Di Psichiatr..

[28] Monell E., Clinton D., Birgegård A. (2018). Emotion dysregulation and eating disorders—Associations with diagnostic presentation and key symptoms. Int. J. Eat. Disord..

[29] Strahler J., Wachten H., Neuhofer S., Zimmermann P. (2022). Psychological correlates of excessive healthy and orthorexic eating: Emotion regulation, attachment, and anxious-depressive-stress symptomatology. Front. Nutr..

[30] Gilmartin T., Dipnall J.F., Gurvich C., Sharp G. (2024). Identifying overcontrol and undercontrol personality types among young people using the five-factor model, and the relationship with disordered eating behaviour, anxiety and depression. J. Eat. Disord..

[31] Obeid N., Valois D.D., Bedford S., Norris M.L., Hammond N.G., Spettigue W. (2021). Asceticism, perfectionism and overcontrol in youth with eating disorders. Eat. Weight Disord. EWD.

[32] Christian C., Bridges-Curry Z., Hunt R.A., Ortiz A.M.L., Drake J.E., Levinson C.A. (2021). Latent profile analysis of impulsivity and perfectionism dimensions and associations with psychiatric symptoms. J. Affect. Disord..

[33] Yung J.J., Tabri N. (2022). The association of perfectionism, health-focused self-concept, and erroneous beliefs with orthorexia nervosa symptoms: A moderated mediation model. Int. J. Eat. Disord..

[34] Frayn M., Livshits S., Knäuper B. (2018). Emotional eating and weight regulation: A qualitative study of compensatory behaviors and concerns. J. Eat. Disord..

[35] Gortat M., Samardakiewicz M., Perzyński A. (2021). Orthorexia nervosa—A distorted approach to healthy eating. Ortoreksja—Wypaczone podejście do zdrowego odżywiania się. Psychiatr. Pol..

[36] Meyer C., Taranis L., Goodwin H., Haycraft E. (2011). Compulsive exercise and eating disorders. Eur. Eat. Disord. Rev..

[37] Auerbach R.P., Mortier P., Bruffaerts R., Alonso J., Benjet C., Cuijpers P., Demyttenaere K., Ebert D.D., Green J.G., Hasking P. (2018). WHO World Mental Health Surveys International College Student Project: Prevalence and distribution of mental disorders. J. Abnorm. Psychol..

[38] Achtziger A., Bayer U.C. (2013). Self-control mediates the link between perfectionism and stress. Motiv. Emot..

[39] Oberle C.D., Watkins R.S., Burkot A.J. (2018). Orthorexic eating behaviors related to exercise addiction and internal motivations in a sample of university students. Eat. Weight Disord..

[40] Augimeri G., Marchese M., Plastina P., Bonofiglio D. (2025). Examining Associations Among Orthorexia Nervosa and Anthropometric Factors and Lifestyle Habits in an Italian University Community. Nutrients.

[41] Istituto Superiore di Sanità (2022). Comunicato Stampa N°05/2022—Disturbi Alimentari: La Prima Mappatura dei Centri del SSN Realizzata dall’ISS.

